# Treated Oil Shale Ashes as Cement and Fine Aggregates Substitutes for the Concrete Industry

**DOI:** 10.1021/acsomega.3c05553

**Published:** 2023-12-04

**Authors:** Sarit Nov, Shay Barak, Haim Cohen, Yaniv Knop

**Affiliations:** † Department of Chemical Sciences, Ariel University, Ariel 40700, Israel; ‡ Department of Chemical Engineering, Ariel University, Ariel 40700, Israel; § Department of Chemistry, Ben Gurion University of the Negev University, Beer Sheva 84105, Israel; ∥ Department of Civil Engineering, Ariel University, Ariel 40700, Israel

## Abstract

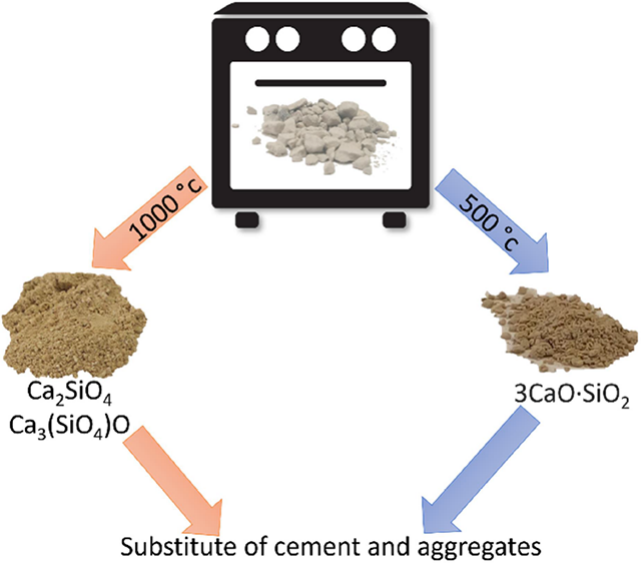

The increased global demand for energy and the environmental concerns associated with fossil fuels highlight the need for alternative approaches. Fossil fuel combustion, particularly coal and oil shale, contributes to greenhouse gas emissions and generates large amounts of ash residues, posing environmental challenges. This study focuses on the potential of thermal treatment to upgrade oil shale bottom ash (OSBA) for use as a cement replacement in concrete, addressing both the economic viability of oil shale combustion and the environmental issue of ash waste management. The findings have significant implications for improving the economics and environmental sustainability of oil shale combustion in construction. By enhancing the properties of OSBA, this study contributes to the advancement of greener energy solutions and waste management practices in the energy and construction sectors.

## 1 Introduction

One of the world’s key economic dilemmas today is the growing energy demand, juxtaposed with the problematic reality that the primary fuels in use—fossil fuels—are major contributors to the greenhouse effect. Mainly due to carbon dioxide emissions as a product of the combustion process and methane leaks via the cycle of natural gas as a cleaner source for power production. Moreover, the process of fossil fuel combustion produces large amounts of ash as waste residues, thus creating a secondary environmental problem that might contaminate the land and aquifers due to the leaching of trace elements.^1,2^ While green energy currently stands as the world’s focal point, in the forthcoming decades, fossil fuels will continue to play a significant role as a global energy source. Among these, coal and oil shale generate substantial ash residues. Notably, oil shale combustion yields significantly higher ash quantities per mass unit than coal, coupled with a markedly lower calorific value. However, oil shale is a much more abundant potential fossil fuel worldwide.^3^ Produced in situ from oil shale, this nontraditional energy source has functioned as an alternative, generating around 400 billion tons of oil on a global scale—exceeding the output of any traditional crude oil source, which is estimated to be over 300 billion tons.^4,5^ As previously stated, oil shale combustion leads to significantly higher emissions of pollutants compared to other fossil fuels. This includes substantial amounts of ash residue. Consequently, the global utilization of oil shale as an energy source remains relatively limited.^6^

Oil shale can potentially be the primary indigenous fossil fuel energy source in the State of Israel, surpassing even the extensive natural gas reserves in the Mediterranean Sea.^7^ These oil shale deposits are distributed across various regions, extending from the Negev Desert in the south to the Zebulun Valley in the north.^8^ In its capacity as a fossil fuel energy source, oil shale manifests as porous stones abundant in organic content. The predominant organic component is kerogen,^6^ constituting a substantial portion with high molecular weight. Oil shales can be used via direct combustion for steam and energy (electricity) production.^9,23^ Apart from being fossil fuels, which contribute to the greenhouse effect, their drawbacks extend to a comparatively low calorific value. This is attributed to the elevated proportion of inorganic material, responsible for the formation of substantial amounts of ash-shaped mineral waste after combustion. Proper treatment is essential for the safe storage of this waste.^10^ Due to the elevated expenses associated with mining and extracting oil shale in contrast to crude oil, only a limited number of oil shale deposits are employed for direct combustion.^11^ Consequently, in situ treatment to generate oil and combustible gases through fracking is a more prevalent approach.^12^

In Israel, significant R&D efforts have been directed toward exploring the feasibility of utilizing indigenous oil shale as a fossil fuel.^13^ Nevertheless, the determination was made that economically, the burning of indigenous oil shale cannot rival coal or natural gas as fossil fuels. Consequently, Israel has not adopted the use of oil shale as a fossil fuel. In Israel, the only industrial company that uses direct combustion of oil shale is Rotem Amfert Negev Ltd.,^14^ which produces phosphate-based products; it is in the Negev desert in Southern Israel, where both oil shale deposits and phosphate rocks are present.^15^ This company employs sulfuric acid to dissolve mined phosphate rock, extracting phosphoric acid from the slurry. The obtained phosphoric acid is then utilized to produce various phosphate-based materials. To access phosphate rock deposits, Rotem Amfert Negev Ltd. must remove the upper layers, which predominantly consist of oil shales consequently, there is no additional expense in the production of oil shale as a fuel. The company employs a 30 MW drop tube boiler to generate steam from the heat produced, and this steam is utilized as a commodity within the plant. Two types of ashes are formed in the combustion process: oil shale fly ash-OSFA (∼10 wt %) and oil shale bottom ash-OSBA (∼90 wt %).^14,16,22^ In Israel, fly ash finds application as a substrate for barn linings and in products designed to absorb animal excrement. The OSBA material produced is stored in large piles (more than 8 million tons) within the industrial plant’s abandoned wastes. Currently, there is no utilization method for the bottom ash, the primary waste residue produced. Thus, large piles of bottom ash are stored under open air near the plant and this fact causes these OSBA piles to be of an environmental concern. A potential utilization can rely on the high content of metals (e.g., iron, magnesium, manganese, copper, aluminum, etc.), thus it can be used as slag replacement for cement production.^20^

About 90% (by weight) of the solid byproducts coming from the production of iron and crude steel are slags. The slag composition includes silica, calcium oxide, magnesium oxide, aluminum, and iron and are the result of removing impurities from the molten steel. According to the World Steel Association (2016, 2018), more than 400 million tons of slag are produced annually worldwide by the steel or iron industry. Steel slag is generally classified by the type of furnace in which it is produced. The characteristic of the slag depends on the type of process used to produce the crude steel, the cooling conditions of the slag, and the evaluation process. Slag can be used in many applications, including as a partial replacement for cement.^21^ In most cases, the use of the byproduct of the steel industry slag, prevents landfilling and contributes to reducing energy consumption, reducing CO_2_ emissions, and helping to preserve natural resources.^17^

This study examines the possibility of upgrading the OSBA as a cement replacement in concrete by performing thermal treatment to the ash. The success of such treatment to improve the quality of oil shale ash to be used not as fine aggregates but rather as a cement substitute can appreciably improve the economics of oil shale combustion as the price of cement is much higher than the price of fine aggregates. In addition, it might help in solving the environmental problem of waste residue treatment of oil shale combustion.

## 2 Experimental Section

### 2.1 Materials

The Oil Shale Bottom Ash (denoted as OSBA) was supplied by ROTEM AMFERT Co.

### 2.2 Analysis and methods

SEM analysis: The ash samples were analyzed in a Ultra-High Resolution Maia 3 FE-SEM microscope by Tescan Company at the Surface laboratory of Ariel University.

XRD analysis: The ash samples were analyzed in an X’pert Pro X-ray diffractometer by PANalytical Company at the Surface laboratory of Ariel University.

TGA analysis: The ash samples were analyzed in a GC100, TGA/DSC 1.STAR by Mettler Toledo Company.

Tube oven: The ash samples were heated in a Quartz ampule in an M.R.C. company tube oven with a 2216L Eurotherm Controls temperature controller.

## 3 Results and Discussion

### 3.1 Untreated OSBA

**Figure 1:**
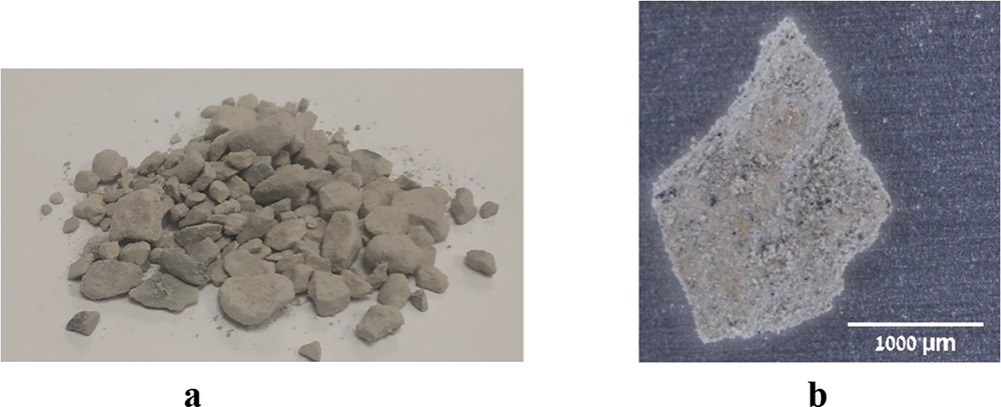
Untreated OSBA. (a) Pile of the large ash particles. (b) Aggregation of small 1500–2000 μm particles forming a larger particle. (Taken from Nov et al.^18^).

The OSBA is consisted of gray particles in the range size 20–50 mm, which are formed by the aggregation of small 1500–2000 μm smaller particles, a photograph of a pile of the untreated OSBA is presented in Figure 1. In addition, the mineral composition of its constituents has been determined via XRD analysis (Table 1).

**Table 1:** XRD Analysis of Untreated OSBA (Taken from Nov et al.^18^)

the component	untreated OSBA [W%]
tricalcium silicate oxide – Ca_3_(SiO_4_)O	40
ettringite – Ca_6_Al_2_(SO_4_)_3_(OH)_12_·26H_2_O	12
calcite – CaCO_3_	32
anhydrite – CaSO_4_	9
potassium nitrate (III) – KNO_3_	4
hydrotalcite – Mg_6_Al_2_CO_3_(OH)_16_·4H_2_O	2
magnetite – Fe_3_O_4_	1

As can be clearly seen, the main constituents of OSBA are calcite CaCO_3_, and tricalcium silicate oxide Ca_3_(SiO_4_)O. The calcium product of the oil shale combustion (carried at 800–100 °C) is lime, CaO. However, during the long-term storage, the lime is transformed into calcite via a direct reaction of the lime with atmospheric carbon dioxide:
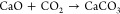
1

**Figure 2:**
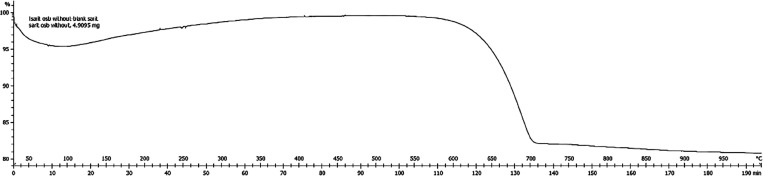
TGA analysis for untreated OSBA^*^ (Taken from Nov et al.^18^). ^*^ Sample mass 4.9 mg; heating rate 5 °C/min.

In order to test the thermal stability under the air atmosphere of OSBA, a thermogravimetric (TGA) analysis in the temperature range 30–1000 °C, at a heating rate 5 °C/min was performed of the untreated OSBA (Figure 2).

The weight changes that occurred during the heating of untreated OSBA show a decrease of ∼4 wt % at RT-105 °C range due to moisture evaporation and mass increase of ∼3 wt %, at the temperature range 100–400 °C, which is probably the result of atmospheric oxygen absorption by the ash to produce mineral oxides. In addition, an appreciable decrease in mass of 17.55% at the temperature range of 550–750 °C is observed. The decrease in mass is attributed to the process of decomposition of minerals in the OSBA, probably, thermal decomposition of calcite, CaCO_3_, via:
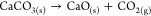
2

Assuming that the calcite is the only decomposing mineral, it is calculated that the calcite content in the OSBA is 39.89 wt %, which is in excellent agreement with the mineral analysis of the OSBA via XRD, which is 40.0 wt %.

### 3.2 Treatment of OSBA

Samples of OSBA were heated in a tube oven for different periods of time to temperatures of 500, 1000, and 1200 °C, and the loss of mass due to the heating was measured (Table 2). The post-heating mass loss percentage of the ash is also shown in Table 2.

**Table 2:** OSBA Mass Loss Percentage Depending on the Thermal Treatment

	mass before heating [g]	mass post-heating [g]	mass loss [%]	temperature [°C]	heating time [h]
1	2.4363	2.2187	8.932	500	2
2	2.7367	2.1365	21.932	1000	2
3	2.7217	2.0558	24.467	1200	2
4	2.3447	2.1368	8.867	500	4
5	2.8778	2.2306	22.490	1000	4
6	2.7043	2.0548	24.017	1200	4

The heat treatment (Table 2) is concerned with 2 or 4 h at 500 or 1000 °C of the thermally treated ash, and the TGA results are within 20 min (the heating time from 600 to 700 °C) and thus definitely decomposition reactions occurring at the longer isothermal heating period will result in different changes in the mass losses. The results, Table 2, indicate that the mass loss of the thermally treated OSBA is very much dependent on temperature comparing 500 to 1000 °C, from ∼9 to ∼22%, but further increase to 1200 °C did not change too much, from ∼22 to ∼24%. In addition, increasing the heating period from 2 to 4 h did not affect the mass loss.

The thermally treated OSBA samples were analyzed via XRD analysis to determine its mineral composition (Table 3).

**Table 3:** Composition of Thermally Treated OSBA

weight percentage %	500 °C, 2 h	1000 °C, 2 h	500 °C, 4 h	1000 °C, 4 h
alite – 3CaO·SiO_2_	61	21.2	56	27
calcite – CaCO_3_	32	10.1	33	1
anhydrite – CaSO_4_	7	8.1	8	4
dicalcium silicate – Ca_2_SiO_4_		24.2		28
hatrurite – Ca_3_(SiO_4_)O		21.2		29
larnite – Ca_2_SiO_4_		13.1		
oldhamite – CaS		2		
quartz – SiO_2_			3	
stishovite – SiO_2_				11

From Table 3, it is clear that in all samples, there is a significant amount of alite, 3CaO·SiO_2_ between 21 and 61 wt %. formed in the treated OSBA. In addition, when the OSBA is heated to a higher temperature, the amount of calcite, CaCO_3_ decreases and this indicates that the calcite decomposes when the temperature rises up. Calcite decomposes to give calcium oxide, CaO. From Table 3, calcium oxide, CaO was not found, so it can be concluded that the calcium oxide reacted with silicate to give alite, 3CaO·SiO_2_, larnite, Ca_2_SiO_4_, or hatrurite, Ca_3_(SiO_4_)O.

The samples were subjected to a lengthier heating process at a lower temperature, leading to an amplified production of alite. The alite is one of the main components in the cement. Therefore, it is expected that, with the advanced process of heat treatment for a longer time at a lower temperature, an improved upgraded oil shale was produced to be used as a component in industrial concrete mixtures.

As also can be seen from the results shown in Table 2, heating the sample to 1000 °C had no significant change of the mass loss of calcite between heating 2 and 4 h. However, as can be seen in Table 3, the weight percentage was significantly reduced. It can be assumed that calcite reacted chemically and did not only decompose. Therefore, the weight loss percentage did not match the XRD results.

### 3.3 SEM Analysis

**Figure 3:**
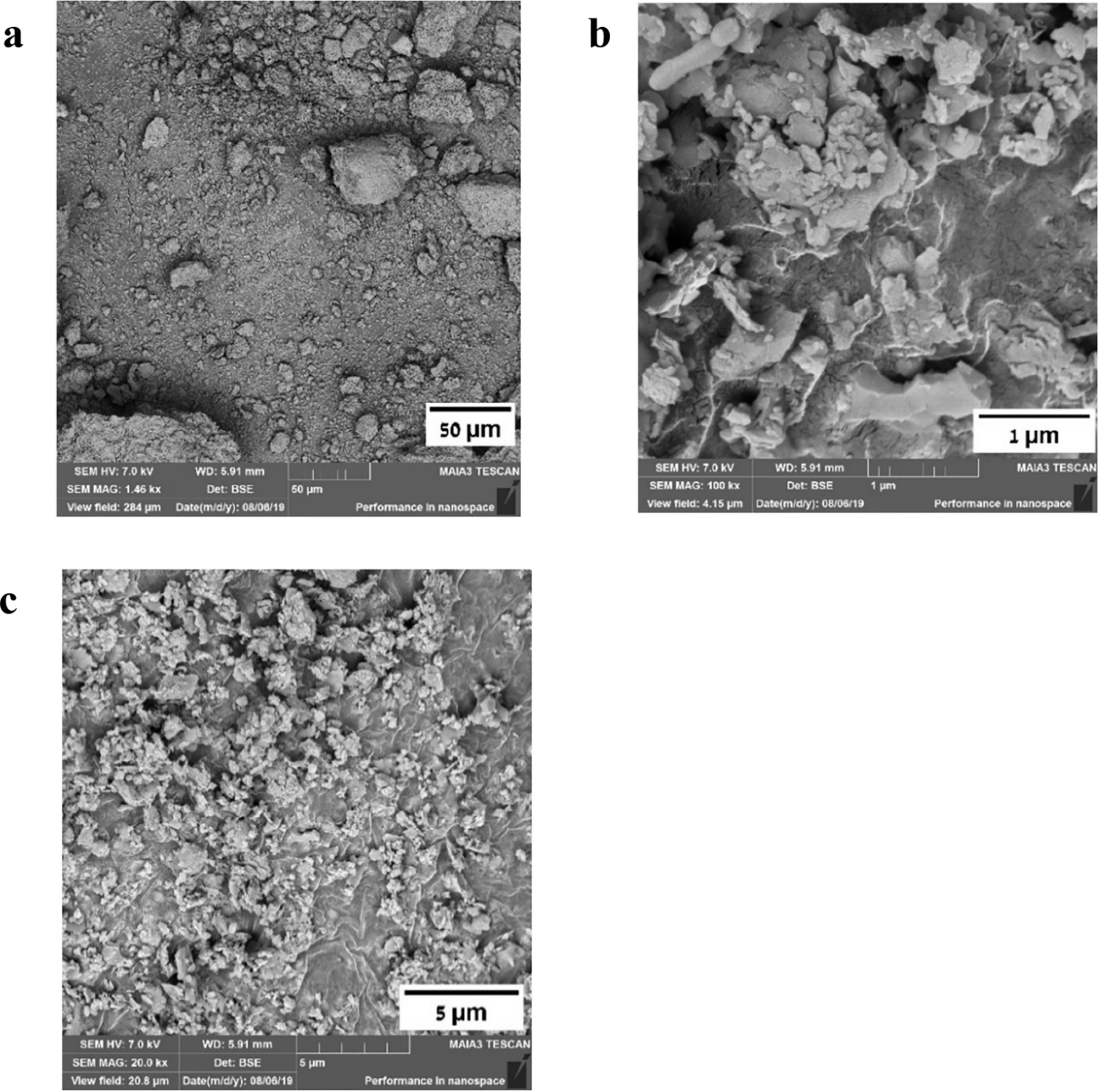
SEM images of nontreated OSBA at different resolutions: (a) SEM amplification, 50 μm. (b) SEM amplification, 1 μm. (c) SEM amplification, 5 μm (taken from Nov et al.^19^).

**Figure 4:**
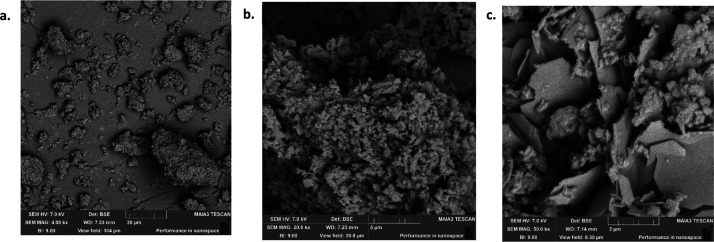
SEM images of OSBA heated for 2 h at 500 °C under different resolutions: (a) SEM amplification 20 μm. (b) SEM amplification 5 μm. (c) SEM amplification 2 μm.

**Figure 5:**
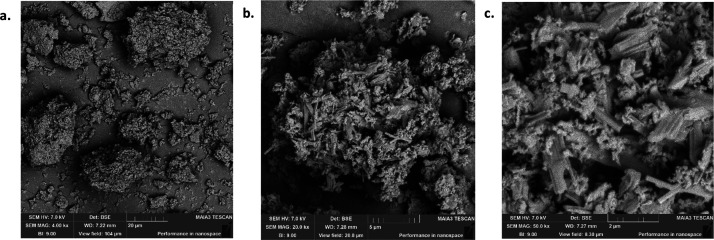
SEM images of OSBA heated for 2 h at 1000 °C under different resolutions: (a) SEM amplification 20 μm. (b) SEM amplification 5 μm. (c) SEM amplification 2 μm.

**Figure 6:**
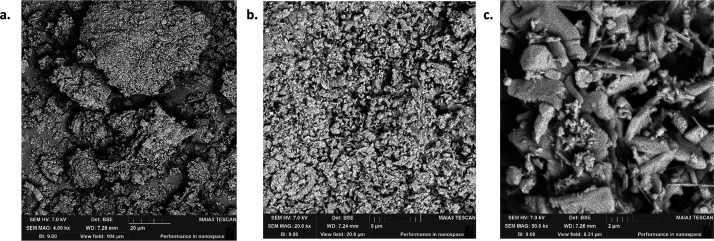
SEM images of OSBA heated for 2 h at 1200 °C under different resolutions: (a) SEM amplification 20 μm. (b) SEM amplification 5 μm. (c) SEM amplification 2 μm.

**Figure 7:**
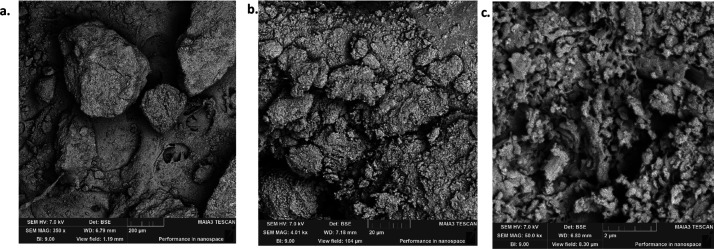
SEM images of OSBA heated for 4 h at 500 °C under different resolutions: (a) SEM amplification 200 μm. (b) SEM amplification 20 μm. (c) SEM amplification 2 μm.

**Figure 8:**
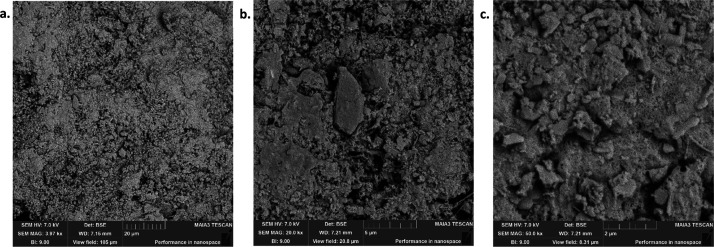
SEM images of OSBA heated for 4 h at 1000 °C under different resolutions: (a) SEM amplification 20 μm. (b) SEM amplification 5 μm. (c) SEM amplification 2 μm.

**Figure 9:**
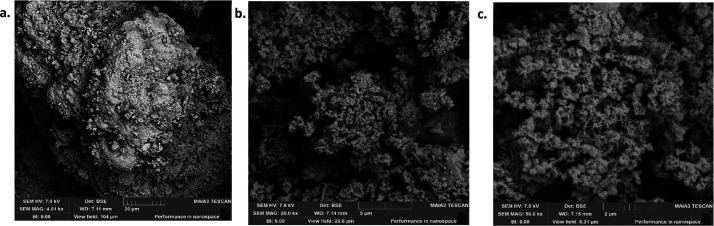
SEM images of OSBA heated for 4 h at 1200 °C under different resolutions: (a) SEM amplification 20 μm. (b) SEM amplification 5 μm. (c) SEM amplification 2 μm.

In addition, SEM analysis of the nontreated OSBA, compared to the treated OSBA samples under various conditions, was carried out and the images are given in Figures 3–9, respectively.

### 3.4 Utilization of Treated Oil Shale Ash in Concrete Blends

The treated oil shale ash was used as a partial substitute for cement in concrete. The blended cement containing a mixture of pure cement and thermal-treated oil shale ash was tested for the production of concrete blends. Cement pastes were prepared and tested according to EN-197 to evaluate the effect of oil shale ash as a partial replacement of pure cement. The cement used is CEM I 52.5 R. All the samples were cured in a water temperature of 21 ± 3 °C.

The mix design of the concrete is presented in Table 4.

**Table 4:** Composition of the Tested Mix Design

material	amount [g]
cement CEM I 52.5 R	450
standard sand	1350
water	225

In order to study and test the effect of oil shale, 50 g of cement was replaced with 50 g of treated oil shale ash. The mixtures were prepared with a constant water-to-cement ratio, and the compressive strengths were measured according to the EN-197.

The properties of the hardened concrete were tested; compressive strengths were determined after 1 day and 28 days post-casting. The result of the compressive strength value determined is the average value of four samples.

**Figure 10:**
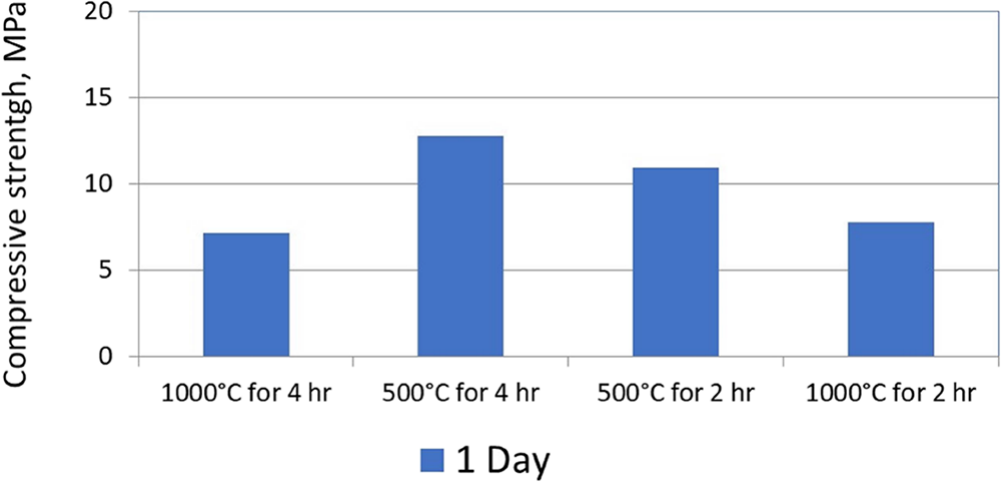
Compressive strength after 24 h from casting using oil shale ashes treated thermally at different temperatures.^10^^*^ Composition of the tested mix design is given in Table 4.

Figure 10 presents the compressive strengths after 24 h from casting. As can be seen, the thermal treatment of the oil shale ash at a lower temperature obtained improved concrete with increased compressive strengths (both for 2 h and for 4 h of oil shale ash thermal treatment). However, after thermal treatment at 500 °C, a longer time of treatment: 4 h, obtained improved strength compared to 2 h of thermal treatment. From Table 3, it is clearly seen that at a lower temperature, the sample contains higher amounts of calcite and anhydrite. The fine calcite powder in the sample can act as nucleation centers and, therefore, increase the rate of the hydration reaction of the cement with water, as was described by Knop et al.^24^ In addition, the increased amount of anhydrite can also be a reason for the increased compressive strength because of the faster hydration of the anhydrite with water. After 4 h of thermal treatment at 500 °C, the amount of both anhydrite and calcite were at the highest level and, thus, the maximum compressive strength was obtained accordingly.

**Figure 11:**
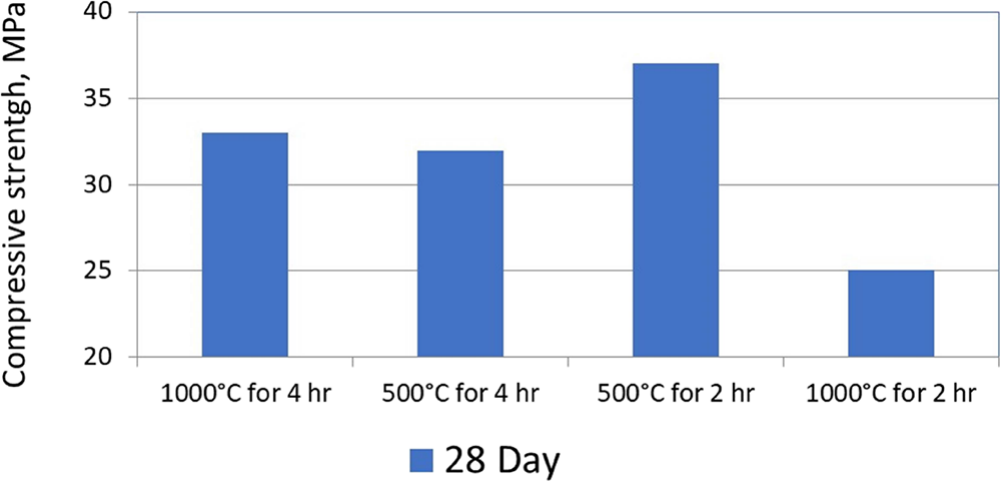
Compressive strength after 28 days from casting using oil shale ashes treated thermally at different temperatures.^11^^*^ The composition of the tested mix design is given in Table 4.

Figure 11 presents the compressive strengths after 28 days from casting. As can be seen, no significant difference in the compressive strengths can be seen between concrete prepared using thermally treated for 4 h at 500 °C or at 1000 °C. However, when using thermally treated oil shale ash for 2 h at 500 °C, the compressive strength was increased compared to using thermally treated oil shale ash for 2 h at 1000 °C. From Table 3, it is clearly seen that the larger amount of alite was obtained after the thermal treatment of oil shale ash at 500 °C for 2 h compared to 2 h at 1000 °C. Thus, it is concluded that there is a direct effect of the amount of alite in the upgraded treated oil shale ash on the final compressive strength of the concrete.

From Figures 10 and 11, it can be concluded that the amount of calcite and anhydrite in the thermally treated oil shale ash mostly effect the initial compressive strengths of the concrete. However, the amount of alite in the thermally treated oil shale ash mostly has an effect on the final compressive strengths after 28 days of casting.

## 4 Conclusions

The following conclusions can be stated:

1. Thermal treatment of bottom ash oil shales at 500–1200 °C for several hours improves the quality of the ash so that it can be used as a component substitute to cement and fine aggregates in concrete mixtures producing improved compressive strength to the concrete.
2. Thermally treated bottom oil shale is a commodity that has economic value in the concrete industry.
3. The amount of calcite and anhydrite in the thermally treated oil shale ash mostly affects the initial compressive strength of the concrete. However, the amount of alite in the thermally treated oil shale ash mostly affects the final compressive strengths after 28 days of casting.
